# Disentangle beneficial effects of strain engraftment after fecal microbiota transplantation in subjects with MetSyn

**DOI:** 10.1080/19490976.2024.2388295

**Published:** 2024-08-20

**Authors:** Eduard W. J. van der Vossen, Mark Davids, Bas Voermans, Koen Wortelboer, Annick V. Hartstra, Annefleur M. Koopen, Pieter de Groot, Evgeni Levin, Max Nieuwdorp

**Affiliations:** a Department of Experimental Vascular Medicine, Amsterdam UMC location University of Amsterdam, Amsterdam, Netherlands; b Horaizon BV, Delft, The Netherlands; c Department of Vascular Medicine, Amsterdam UMC location University of Amsterdam, Amsterdam, Netherlands

**Keywords:** FMT, strain engraftment, CCA, gut microbiota, microbiome, metabolites

## Abstract

Fecal Microbiota Transplantation (FMT) has emerged as a potential modality for mitigating microbiome-associated diseases. Despite this potential, the precise causal pathways by which specific gut microbiota strains induce remission remain inadequately elucidated. In this study, we aimed to discern the impact of engraftment of donor-infused strains on alterations in plasma metabolites, subsequently contributing to the amelioration of clinical parameters involved in subjects with metabolic syndrome (MetSyn) receiving an FMT. We observed that a higher fraction of donor strains engrafted in the recipient is correlated to a reduction in diastolic blood pressure and found specific strain associations through canonical correlation analysis. Integrating the metabolomics profile shows that engraftment of *Collinsella aerofaciens* and *Fusocatenibacter saccharovorans* was related to a reduction in 2-oxoarginine in plasma, which was subsequently correlated to a reduction in diastolic blood pressure. In conclusion, we applied a novel framework to elucidate on the complex and heterogenous FMT intervention, establishing a connection between engrafted microbiota and clinical outcome parameters. Our findings underscore the potential therapeutic efficacy of FMT in ameliorating MetSyn, demonstrating a potential contribution of microbial strain engraftment to the improvement of MetSyn via modulation of circulating metabolites.

## Background

Ever since the astounding cure rate of over 90% in subjects with recurrent *Clostridiodes difficile* (rCDI) infection,^1^ fecal microbiota transplantation (FMT) has been widely studied in an attempt to understand and alleviate the burden of different types of diseases. FMT is a procedure in which feces from either a healthy lean donor (allogenic) or own feces (autologous) are infused in a recipient with the disease under study. The mechanism of action of an FMT has yet to be determined. In rCDI patients, the high success rate is attributed to the donor gut microbiome which engrafts and outcompetes *C. difficile*,^2,3^ which is substantiated by the observation of increased microbial diversity in the stool of rCDI patients after receiving an FMT.^1^ It is postulated that the FMT procedure resolves any disease that arises due to a dysbiosis as it restores the microbial diversity via strain engraftment.^4^ However, in more complex pathophysiological ailments and clinical conditions, such as metabolic syndrome (MetSyn), the clinical success rate is lower.^5^

To better understand the relationship between microbial strain engraftment in an FMT and clinical success, a multitude of FMT studies for a wide range of clinical conditions were investigated.^6–8^ Critical determinants influencing the success of strain engraftment include the propagule pressure, the method of delivery, the infectious nature of the disease, and the administration of antibiotics. However, a discrepancy persists between the rate of donor strain engraftment and the clinical efficacy of the intervention. This incongruence may be ascribed to the heterogeneity of the recipient microbiome state before the intervention, the heterogeneity in composition of the donor microbiome introduced, the applied protocol, and the diverse range of diseases under investigation. From a microbiome perspective, alteration in gut microbiome due to active treatment, e.g., the engrafting microbiota, is accompanied with changes metabolic function.^9^

Given its greater complexity than recurrent Clostridiodes difficile infection (rCDI) from a pathophysiological perspective and its clinical significance attributable to the escalating global prevalence,^10–12^ MetSyn emerges as an appropriate target population for elucidating the intricate interplay between FMT and pertinent clinical parameters. MetSyn is the constellation of several disorders, including high blood pressure, raised fasting glucose, dyslipidemia, and central obesity.^13^ The state of dysmetabolism is intertwined with a change in gut microbiome, mediated through diet and lifestyle. This led to the rationale that an FMT, which targets the dysbiotic microbiome, could contribute to addressing the prevalent health concerns associated with MetSyn. One study in which clinical success was observed in subjects with MetSyn was the FMT study of Kootte et al. Here, short-term improved insulin sensitivity was accompanied by altered microbiota composition and changes in host plasma metabolites.^14^

To further build on the hypothesis that clinical success can be obtained via alteration in gut microbiota, and mediated through host plasma metabolites, we re-investigate a few studies which attempted to improve MetSyn status through an FMT.^15–17^ Although some of these studies did not show significant clinical remission in glycemic parameters, we investigated the relation of specific engrafted gut microbiota with alterations in clinical parameters related to MetSyn. Rather than viewing FMT as a single intervention, using strain tracking allows for the detailed monitoring of individual microbial species’ engraftment within the recipient’s gut microbiome, providing insights into the specific contributions of each strain to overall health outcomes. We hereafter attempt to describe these engraftments in relation to changes in plasma metabolites to further characterize the complex FMT intervention, which ultimately serves as a novel metric to interpret data from FMT studies.

## Methods

### Clinical study selection criteria and study design

The data used for this investigation were obtained from three studies, called Appetite,^15^ Fatmed^16^ and Febaligo,^17^ and were previously separately published. The baseline metabolomics data sets from these studies were collectively analyzed previously.^18^ Each study had its own study population with, however, overlap as all studies recruited subjects with MetSyn. The specifics of each sub-study, including inclusion- and exclusion criteria, are summarized (Suplementary Table S1). All sub-studies recruited MetSyn subjects using the National Cholesterol Education Program (NCEP) criteria for MetSyn (at least three out of the following five criteria: fasting plasma glucose ≥5.6 mmol/L, triglycerides ≥1.7 mmol/L, waist circumference >102 cm (males) or >88 cm (females), high-density lipoprotein (HDL) cholesterol <1.03 mmol/L (males) or 1.30 mmol/L (females), blood pressure ≥ 130/85 mmHg). Appetite and Febaligo used donors whom had undergone bariatric surgery and lost at least 30% of their pre-surgical weight in the first year after surgery. Furthermore, within the Fatmed study, FMT was applied in combination with a Mediterranean diet.^16^ For all studies, written informed consent was provided for all participants. The procedures of each sub-study were approved by the local Institutional Review Board of the Amsterdam UMC and conducted in accordance with the Declaration of Helsinki. The studies were registered at the Dutch Trial Register and accessible at the International Clinical Trials Registry Platform (https://trialsearch.who.int) with the trial numbers NTR4713, NTR5983, and NTR4327 for the Appetite, Fatmed, and Febaligo, respectively.

### Fecal microbiota transplantation procedure

In all sub-studies, the FMT procedure as described in van Nood et al. was applied with minor modifications.^1^ In short, subjects receiving FMT did not receive antibiotics prior to the intervention. Via gastroduodenoscopy, a duodenal tube was placed with its position verified via X-ray. Dependent on the study (see Supplementary Table S1), varying amounts of dissolved macrogol/electrolytes solution (Klean-Prep) were infused for bowel lavage (usually 3 L). On the day of donor feces infusion, patients were sober. Feces of the donor were collected in the morning prior to the FMT. This was mixed with a saline solution (0.9% NaCl) until fully homogenized. Hereafter, the solution was sieved to remove all debris. The donor feces solution (500 cc in total) was slowly infused with a 50-cc syringe through the duodenal tube.

### Gut microbiome sequencing analysis

#### DNA isolation and purification

Genomic DNA was extracted from 0.25 g of fecal samples using the repeated bead beating method of Yu and Morrison, with the following modifications. Three types of sterile zirconia beads (Thistle Scientific, Glasgow, United Kingdom) were used (0.5 g in total; one 3.0-mm bead, 0.1 g of 0.5-mm beads, and 0.3 g of 0.1-mm beads). Fecal samples were homogenized three times for 60 s at maximum speed on a Mini-Beadbeater-24TM (Thistle Scientific, Glasgow, United Kingdom), with the samples cooled on ice for 60 s in between bead-beating cycles. The supernatants of two bead-beating rounds were pooled and incubated with 350 μl of 7.5 M ammonium acetate (Sigma, St. Louis, MO, United States) on ice. The extraction proceeded as per the RBB protocol using Qiagen’s DNeasy® Blood & Tissue Kit (Qiagen, West Sussex, United Kingdom) according to the manufacturer’s instructions for the final DNA purification (without the lysis steps and eluted in 100 μl of AE buffer).

#### DNA library preparation

Genomic DNA was quantified using the Qubit dsDNA high-sensitivity assay kit (Invitrogen – Carlsbad, California, United States). Samples were prepared for shotgun metagenomic sequencing using the Illumina Nextera XT library preparation kit and following the manufacturer’s instructions. Unique Nextera XT 8-nt dual indices were used for multiplexing (Illumina, San Diego, CA, United States). Libraries were pooled to an equimolar concentration and sequenced by Edinburgh Genomics (Edinburgh, United Kingdom) using a 2 × 150-bp paired-end method on an Illumina NovaSeq 6000 platform and aiming to achieve ∼4–5 Gbp of sequencing data per sample.

#### Plasma metabolites

Profiling of untargeted metabolomics was performed by Metabolon using ultra high-performance liquid chromatography coupled to tandem mass spectrometry (UPLC-MS/SM), as previously described in Koh et al.^19^ The raw output was further normalized to account for batch effects. Furthermore, per metabolite, the median value is scaled to 1, in which missing values were imputed by half of the lowest respective value, to distinguish between the lowest measurable value and values that fell below detection limit.

### Bioinformatic pipelines

#### Gut microbiome and microbiota profiling

The raw reads underwent an initial check and a quality filter using fastp (v.0.20.0).^20^ This tool detected and removed the adapter, trimmed 5 bp from read1, and applied a sliding window for quality trimming using a window width of 4 bp and a Q-score threshold of 15. Reads that were shorter than 70 bp were omitted. The paired-end reads that passed the quality filtering were mapped to the human genome (hg19) using Bowtie 2 (v.2.4.1)^21^ with the very-sensitive setting and the inclusion of dovetail. SAMtools (v.1.15.1)^22^ was used to convert the SAM file to BAM and remove the reads that were mapped to the human genome. Sambamba (v.0.7.1)^23^ was used to sort the unmapped reads by name, with a memory limit of 40 gigabytes. BEDtools (v.2.27.1)^24^ was used to convert the sorted unmapped reads to forward and reversed fastq format. The high-quality, non-human reads were then subsampled to 20 million paired-end reads per sample using seqtk (v.1.3 r106). The forward and reversed reads were concatenated and fed into the HUMAnN3 pipeline (v.3.6).^25^ For each sample, the species genome bin (SGB) level microbial composition was inferred using MetaPhlAn4 (v.4.0.5).^26^ The reads were then mapped against the pangenomes selected based on the inferred composition (using Bowtie 2), and the unmapped reads were translated and mapped against the full UniRef90 protein database using DIAMOND (v.2.0.15). The community-level abundance of the MetaCyc pathways were normalized to copies per million.

#### Strain-level profiling

To ascertain the origin of a specific SGB (donor or recipient before intervention), we applied strain-level profiling using StrainPhlAn 4 (v4.0.5).^27^ Samples were kept if they had at least 20 markers and a marker was kept if it was in at least 50% of the samples. As no consensus exists on the definition of strain, we adopted the method earlier used in Ianiro et al.^7^ This definition includes an operational species-specific definition of “strain” obtained by comparing phylogenetic distance distributions of microbial genetic profiles sampled from potentially related pairs and compared to unrelated individuals.^7^ In short, we extracted pairwise normalized phylogenetic distances from the trees built in StrainPhlAn within our cohorts. We subsequently determined which sample pairs are potentially related by having both a pre- and post-FMT sample. As donor engraftment might take place, SGBs can be replaced by the donor SGB infusion, and SGBs from the donor may be related to SGBs in the recipient after FMT intervention, making these sample pairs also related. We furthermore determined which sample pairs are unrelated as done previously by Valles-Colomer et al.^28^ Hereafter, we estimated the strain-identity thresholds. When there were at least 50 potentially related samples, we used either Youden’s index or the 5th percentile of the unrelated sample pair group (based on which is most conservative). When there were less than 50 related samples present for a given SGB, we used the third percentile of the unrelated sample pair group. We included the baseline dataset of a separate cohort^29^ to get as much SGBs as possible to pass the presence filter for StrainPhlAn. In total, 30 low-prevalence SGBs were omitted as there were too few positive samples. As these low-prevalence SGBs in the FMT cohorts have a negligible impact on the overall results and lack potential for statistical inference, they were considered to have minimal relevance to the overall investigation. To resolve discrepancies in strains within the post-FMT sample shared with both the recipient pre-FMT sample and the donor sample, determined by the predefined threshold, the strain was assigned based on the lowest normalized genetic distance, indicating a closer relationship to either the recipient pre-FMT or donor sample. All SGB information regarding the selected threshold is presented in Supplementary Table S2.

### Definition of FMT triads, assessing strain sharing, and engraftment

As mentioned previously,^7^ an FMT triad is defined as a set of sample combinations, including a pre- and post-FMT sample of the recipient with its donor sample. The strain-sharing rate is defined as the total number of shared strains between two samples divided by the number of species profiled by StrainPhlAn in common between the two samples. The strain-sharing rate per sub-study was visualized by unsupervised networks using the package “igraph” in R (v1.2.6).^30^ The Fruchterman-Reingold layout algorithm was applied, with the number of shared strains as the squared edge weights and the samples representing the nodes. Fraction of strains shared between post-FMT and the donor and post-FMT and pre-FMT is defined as the number of strains shared between the two samples, divided by the number of strains profiled at post-FMT. The subsequent analyses focus on the engraftment of the SGBs in the recipient. Hereby, different groups are created where the division of the “engraftment” group depends on the specific SGB that either shows engraftment or no engraftment in the specific subject.

### Regularized canonical correlation analysis

To integrate two datasets and examine the relationship between engrafted strains and changes in clinical parameters, we employed regularized canonical correlation analysis (rCCA; function ‘rcc’ of the “mixOmics” package).^31^ This approach was chosen to account for the limited sample size while identifying candidate strains for subsequent statistical analyses. We selected strains with the largest engrafting rates or the largest post-FMT relative abundance and used the binary engraftment as an input. For the second dataset, we used the change (post–pre) in clinical parameters. To avoid overfitting due to the low sample size and thereby the relatively large number of variables, we applied Ridge’s penalization (L2 penalty). Ridge parameter λ was estimated using a grid search with LeaveOneOut cross-validation, utilizing the standard grid for λ (function ‘tune.rcc’ of the “mixOmics” package). In more detail, for each combination in the grid, the pairwise correlation is calculated between the scores of each dataset. These scores were computed for each subject within each dataset by multiplying the values of the corresponding original datasets by subject-specific loadings. During the LeaveOneOut cross-validation procedure, one sample was withheld from the analysis in each fold, and the scores were computed based on the remaining samples. This process is iterated until each sample has been left out once. Subsequently, the pairwise correlation coefficient was calculated via the pooled scores of all iterations between the two datasets. After calculating the pairwise correlation coefficient for each combination of the grid, the optimal λ for each dataset was determined as the values in the grid that produced the largest correlation. Hereafter, rCCA was conducted on the full dataset,^32^ and the results were further visualized via the correlation circle plot. Subsequently, the inner product, defined as the product of the two vectors’ lengths and their cosine angle^33^ were calculated to define a subset of interesting linear relations between strain engraftment and changes in clinical parameters. The top 10 largest absolute inner product values were taken for further statistical analyses. Given that rCCA exclusively handles complete cases, we applied linear mixed-effect models for addressing missing data, as described in the section “Statistical analysis”. After finding potential links between strain engraftment and clinical parameters, we included the metabolites that also were significantly related to the engrafted strains.

### Statistical analysis

To assess the degree of strain engraftment between samples, we employed a linear model. The analysis was designed to investigate the dissimilarity between a true donor and a post-FMT recipient. The outcome variable was the dissimilarity, which is defined as the 1 minus the number of shared strains divided by the maximum number of shared strains, in which the maximum number of shared strains means the SGBs that were assessed by StrainPhlAn which occur in both subjects. The two predictor variables were the sample type (either pre- or post-FMT) and donor (either true or false).

To acquire the similarity between the different types of data (gut microbiota, their pathways, metabolites, and metadata), Procrustes analysis was applied to obtain maximum similarity (function ‘procrustes’ of the “Vegan” package). To test for the nonrandomness between the two configurations, we applied a permutation test (function ‘protest’ of the “Vegan” package). The number of permutations done was 999.

To assess the effect of strain engraftment on changes in clinical parameters with consideration for repeated measures, linear mixed-effect models were applied on the top 10 combinations of strain engraftment and change in clinical parameters based on the rCCA method (see “Regularized Canonical Correlation Analysis”). The outcome variable was the clinical parameter and the predictor variables were the specific strain engraftment (binarized), time and its interaction. Subjects were included as a random effect. To determine the appropriate t-distribution and ultimately assign p-values, Satterthwaite’s approximation was applied to calculate the degrees of freedom (function ‘lmer’ of the “lmerTest” package). For all statistical analysis, R version 4.3.0. was applied. All R-scripts are readily available at https://github.com/EvdVossen/MPS.

## Results

**Figure 1.**
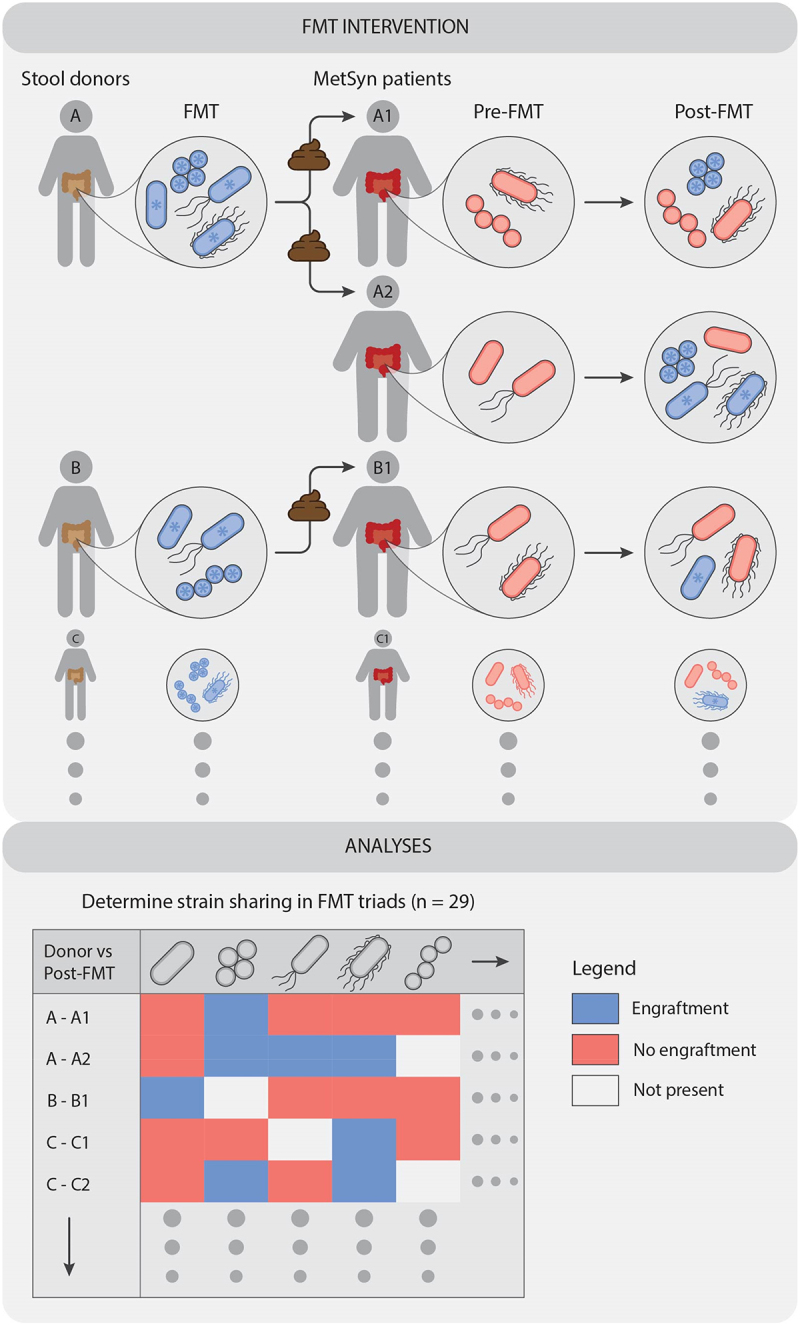
A simplified schematic representation of the process of pitting the donor gut microbiome against the recipient (MetSyn patients) their gut microbiome is presented. This process involves the introduction of many different strains from many different FMT donors, resulting in either engraftment or no engraftment in the recipient after FMT.

**Figure 2.**
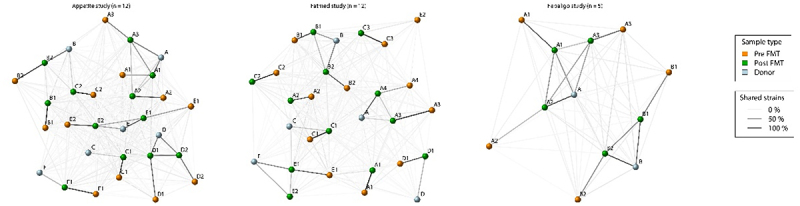
Strain-sharing networks of the different studies. The letters correspond to the specific donor. Subjects receiving FMT from the same donor have the same letter. Additionally, to distinguish between FMT recipients, these subjects received an additional number.Number.The letter and number combinations correspond to specific FMT triads belonging to the specific donor, with the color of the node distinguishing the sample types (orange = pre-fmt; green = post-fmt; blue = donor). The edges represent the percentage of strain-sharing between the different samples.

**Figure 3.**
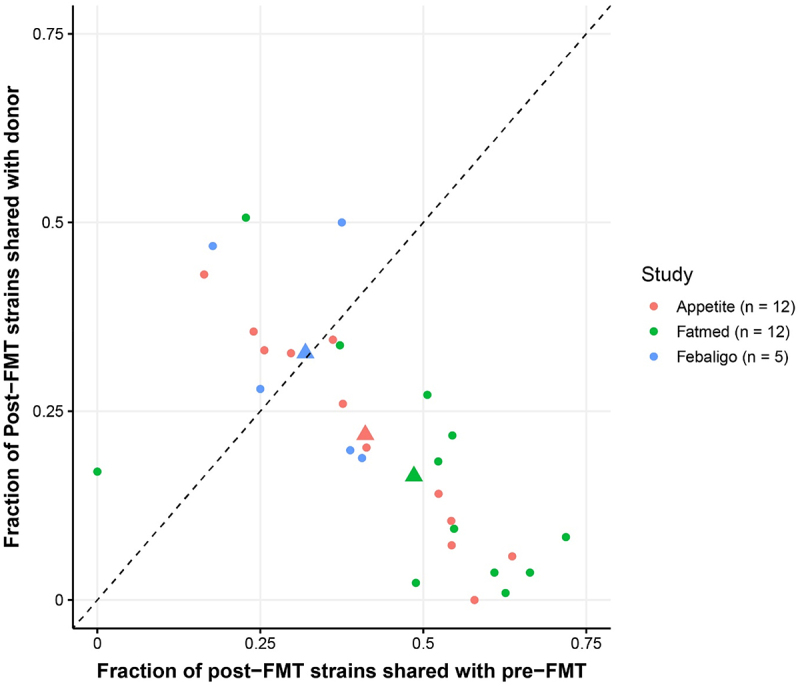
Fraction of donor strains that are shared with the post-fmt sample against the fraction of pre-fmt strains that are shared with the post-fmt within each of the FMT triads. The fraction is defined by the number of shared strains (either between pre- and post-fmt samples or between donor and post-fmt samples) divided by the total number of strains in the post-fmt sample characterized by StrainPhlAn. The larger triangles represent the mean position for each sub-study. The diagonal dashed line represents a cutoff in which post-fmt samples above this line share a larger fraction of strains with its donor and below this line share a larger fraction of strains with its pre-fmt sample.

In total, from the three sub-studies, 29 FMT triads were analyzed to disentangle the complex heterogeneous FMT interventions. The gut microbiota with its microbiome and metabolites were analyzed in relation to clinical parameters (see Methods). At the global ecological level, we examined β-diversity on the whole population and stratified by donor and observed minimal disparities (Suplementary Figure S*1*). These findings suggest that such conventional ecological methods may lack the depth required for comprehensive analyses in an FMT setting. From an intervention standpoint, the introduction of a strain can lead to engraftment or no engraftment. In an FMT setting, the complexity increases even further because there are dozens of strains introduced into the recipient per donor on a strain-by-strain basis (Figure 1). To investigate these more intricate relationships, the strain-sharing rates were assessed per sub-study between the recipient pre- to post-FMT and between the donor and post-FMT and visualized in unsupervised networks (Figure 2). These unsupervised networks clearly show the commonality of strains between pre- and post-FMT, as well as a donor relationship (linear model dissimilarity β_donor_post_ = [0.19, 0.16, 0.27]; *p* = [8.1e^−7^, 3.9e^−6^, 1.7e^−3^]) in all sub-studies (Appetite, Fatmed and Febaligo, respectively). For most samples within the three sub-studies, strains tend to be more retained within the individual as opposed to engraftment of donor strains (Figure 3), which is in line with previous literature.^6,7^ We hereafter used the fraction of strains within the post-FMT sample which originate from the donor and used it as a measure to associate to changes in clinical parameters (Suplementary Table S*3*). Interestingly, for the rate of glucose disappearance and diastolic blood pressure significant correlations were found (ρ = 0.46 and −0.47; *p* = 0.012 and 0.044 for rate of glucose disappearance and diastolic blood pressure, respectively), indicating a link between strain engraftment and improvement in these clinical parameters.

**Figure 4.**
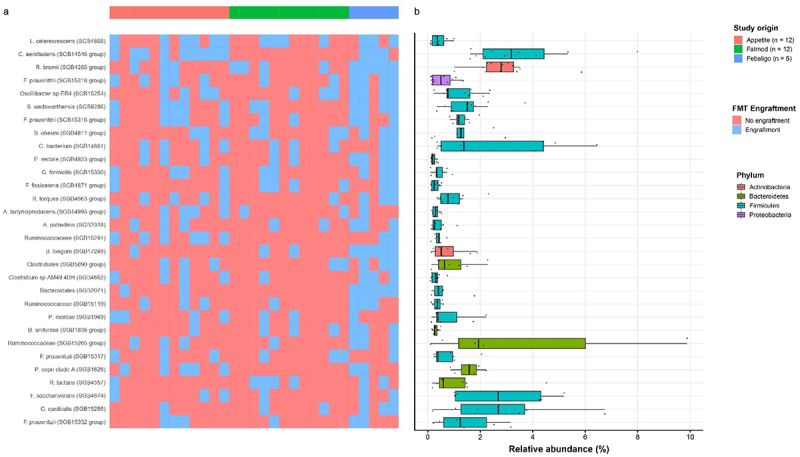
(a) Heatmap depicting the engraftment status of the top 30 most engrafting donor strains (rows) in relation to the subjects separate post-fmt (columns). The subjects were grouped per sub-study depicted by the sidebar on top of the heatmap. Engraftment of the corresponding donor SGB is indicated by the threshold output of StrainPhlAn. (b) Relative abundance of the most engrafting SGBs in post-fmt samples colored by phylum taxonomic level.

To identify specific engrafted gut microbiota that exert the potential beneficial effect within the heterogenous FMT intervention, we investigated the strain transfer from the donor to the recipient post-FMT. This is the number of transferences between the donors and recipients after FMT per strain (Figure 4a). We furthermore investigated the relative abundance after FMT in all subjects having the strain engrafted (Figure 4b). The most transferred strains, among others, included *Lacrimispora celerecrescens* (SGB4868), *Ruminococcus bromii* (SGB4285 group), *Collinsella aerofaciens* (SGB14546 group), *Sutterella wadsworthensis* (SGB9286), *Oscillibacter* sp ER4 (SGB15254), multiple *Faecalibacterium prausnitzii* SGBs (SGB15316 group, SGB14317 group and SGB15318 group), *Gemmiger formicilis* (SGB15300), and *Eubacterium rectale* (SGB4933 group). Interestingly, *Prevotella copri* Clade A (SGB1626) was transferred in a limited number of cases from the donor to the recipient following FMT. However, it exhibited considerable relative abundances post-FMT. This is further observed when directly comparing the relative abundances before- and after the intervention (Suplementary Figure S2). We observe different engraftment patterns when comparing the *P. copri* Clade A (SGB1626), *R. bromii* (SGB4285 group), and *C. aerofaciens* (SGB14546 group). Engraftment of *P. copri* Clade A (SGB1626) only occurred in subjects which did not have a native strain prior to the intervention and occupation of large a niche present in the gastro intestinal tract, which is occupied after the FMT intervention. Interestingly, *R. bromii* (SGB4285 group) does show a significant increase in relative abundance after the intervention (*p* = 0.032), which indicates an expanded niche for the engrafted strain compared to the native strain. The *C. aerofaciens* (SGB14546 group), on the other hand, is showing signs of replacement or co-occurrence in a fixed niche as there is no increase in relative abundance. This further highlights the heterogeneity and complexity of the FMT as an intervention.

**Figure 5.**
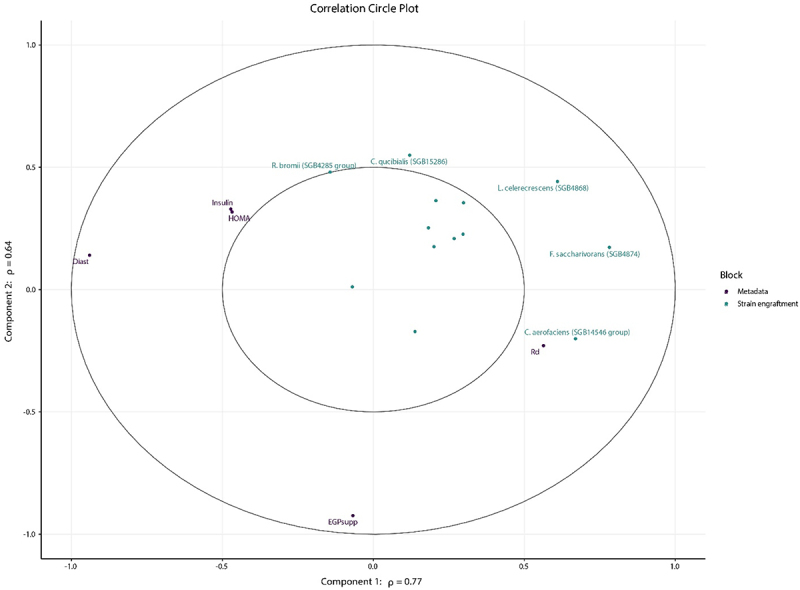
Correlation circle plot depicting the first two canonical variates with coordinates of the different variables of the strain engraftment- and clinical data modalities in the same dimensional space. The coordinates of each feature were obtained via the correlation between each original (centered and scaled) feature and each of the canonical variate. Note that this analysis included a total of 19 subjects rather than 29 as 10 subjects from the Fatmed study had missing data in diastolic blood pressure values.

To disentangle the effects of the FMT intervention on the clinical outcome, we applied rCCA. As an input, we used the binary strain engraftment and a sub-selection of clinical parameters after baseline correction (post-FMT values minus pre-FMT values). The clinical parameters selected were based on the correlation between the fraction of strains in the post-FMT samples that were shared with the donor (Suplementary Table S*3*). As blood pressure has a strong relation with the gut microbiome,^34^ we used the subjects with complete data on diastolic blood pressure. Furthermore, as the number of subjects for the rCCA is small relative to the number of features, we applied ridge regularization. The optimal parameters for each dataset ($\lambda_{1}$ and $\lambda_{2}$) were found via a grid search (Supplementary Figure S3A) and the correlations of the canonical variates were determined (Supplementary Figure S3B). We visualized the first two canonical variates and plotted the potentially interesting features of both the engrafted strains and clinical parameters, defined by its presence in the outer ring of the correlation circle plot (Figure 5). We then applied a linear mixed-effect model for the 10 combinations with the largest absolute inner product.^33^ Most notably, a negative correlation between the engraftment of *C. aero-faciens* (SGB14546 group) and the delta blood pressure (coefficient of −16.0 mmHg; FDR-corrected *p* = 1.11·10^−2^) was observed. Interestingly, *Fusicatenibacter saccharivorans* (SGB4874) and *L. celerecrescens* (SGB4868) showed a similar relation to blood pressure, which is in part explained by the large overlap in engraftment of this species in the FMT recipients (coefficients of −19.5 and −13.2 with FDR-corrected p of 3.96·10^−3^ and 3.52·10^−2^, for *F. saccharivorans* (SGB4874) and *L. celerecrescens* (SGB4868), respectively).

Next, we investigated a link between the engrafted strains, changes in metabolites and clinical parameters. Initially, we established significant relationships between the gut microbiota, metabolites, and the metadata based on the Procrustes analyses (Supplementary Table S*4*). Thus, on a global level, the gut microbiota and circulating metabolites are significantly correlated, setting the ground to find direct relationships between strain engraftment and metabolites. We also established a relationship between the metabolites and diastolic blood pressure based on multilevel PCA (Supplementary Figure S*4*; *p* = 0.024). To identify specific relationships between diastolic blood pressure and metabolites, we conducted a univariate correlation analysis (Supplementary Figure S*5*).

Next, we identified metabolites that are associated with both clinical parameters, as well as the engraftment of *C. aerofaciens* (SGB14546 group) and *F. saccharivorans* (SGB4874). We found 2-oxoarginine (also named α-keto-δ-guanidinovaleric acid) to significantly decrease in subjects having engraftment of the aforementioned strains (*p* = 0.048 and 0.001 for *C. aerofaciens* and *F. saccharivorans*, respectively), as well as a positive correlation between the change in 2-oxoarginine and the change in diastolic blood pressure (ρ = 0.46, *p* = 0.049). As 2-oxoarginine is part of the arginine dehydrogenase pathway, another intermediate within this pathway, named 4guanidinobutanoate, was investigated. Interestingly, a similar pattern is observed (Supplementary Figure S6). Thus, reduction of these metabolites in the plasma is concomitant with the engraftment of the strains.

We also found biliverdin to be significantly associated with *C. aerofaciens* engraftment (*p* = 0.016) and a negative correlation between the change in biliverdin and the change in diastolic blood pressure (ρ = −0.5, *p* = 0.028). Given that biliverdin is an intermediate from heme to the production of bilirubin,^35^ which is also considered to be related to blood pressure via increasing the availability of nitric oxide (NO),^36^ we also investigated bilirubin. We did, however, not find any correlation to blood pressure in our dataset.

## Discussion

In the context of FMT studies, standard ecological measures such as the β-diversity has been shown to be insufficient in capturing the complexity of an FMT intervention. We therefore employed a novel framework to disentangle the complex heterogeneous FMT intervention in subjects with MetSyn. From an intervention standpoint, each engrafted gut microbiota component can be viewed as an intervention. Hence, FMT, by its inherent nature, may be considered a distinct intervention for individual subjects. Various strains engraft uniquely in each recipient, potentially altering diverse pathways leading to clinical remission. It is therefore of importance to first identify key strains that engraft and potentially exert a beneficial effect. Within our three sub-studies, we observe that the fraction of strains shared between the post-FMT sample and pre-FMT sample is predominantly more frequent than the fraction of strains shared between the post-FMT sample and the donor. This is in line with previous meta-analyses papers published, in which key driving factors for a larger strain engraftment rate from the donor are based on characteristics such as using both the upper- and lower route of delivery, the use of antibiotics, and the fact that the disease is infectious.^6,7^ Thus, from an ecological point of view, the host microbiome state in our cohort is likely simply not as deteriorated, leading to resistance in engraftment.

Strikingly, we found that a number of donor strains in the recipient post-FMT correlated with a reduction in diastolic blood pressure as well as a faster rate of glucose disappearance. This suggests that, in general, strains originating from the donor are related to improvement of the MetSyn status, supporting the hypothesis that the microbes infused lead to restoration of the microbial diversity.^4^ Interestingly, we discern a relationship between specific engrafting strains and diastolic blood pressure. However, this phenomenon is not observed concerning the rate of glucose disappearance. This observation suggests that associations between the rate of glucose disappearance and the microbiome are likely to manifest at an ecological level rather than with specific taxa. Of note, it is important to mention that the decrease in diastolic blood pressure is very large, outperforming common antihypertensive drugs. Part of this decrease can likely be attributed to a predisposed high blood pressure caused by the stress of being in clinic, often referred to as the “white coat hypertension”.^37^ Furthermore, potential placebo-related factors could also lead to the decrease in diastolic blood pressure on top of the more subtle effect of the FMT.^38^

Within the interventions, different engraftment patterns are observed, varying from replacement or co-existence with increase in abundance (*R. bromii*) or without (*C. aerofaciens*) to novel engraftment (*P. copri* clade A). It is worth to note that 80% of *C. aerofaciens* strains possess the N-acetylgalactosamine utilization pathway.^39^ This pathway enables these strains to utilize N-acetylgalactosamine, a component of the A-antigen found in intestinal mucin. By utilizing this substrate, these strains potentially gain a competitive advantage. Moreover, this pathway in general is linked with higher α-diversity and improved cardiometabolic health in individuals with the A-antigen.^39^ Using strain engraftment as an input for the rCCA showed a correlation between both *C. aerofaciens* (SGB14546 group) and *F. saccharivorans* (SGB4874) engraftment and changes in diastolic blood pressure. Unexpectedly, we did not find any significant correlation between engraftment of *R. bromii* and blood pressure dynamics. *R. bromii* is considered a keystone species for the breakdown of resistant starch, and resistant starch is related to lowering of blood pressure.^40,41^

Next, we investigated which metabolites could serve as an intermediate between microbial fermentation and clinical outcome. 2-oxoarginine was found to be one of the metabolites which could be linked as an intermediate within the metabolon panel, connecting blood pressure drop to strain engraftment. The observed relationship between *C. aerofaciens*, *F. saccharivorans*, 2-oxoarginine, and diastolic blood pressure might be explained by a combination of strain and host genetics, for which genetic data are not available in this study.^39^ Interestingly, previously, two of the top engrafted species (*C. aerofaciens* and *F. saccharivorans*) were all enriched in responders to a lifestyle intervention and correlated to arginine biosynthesis.^42^ Indeed, all these species contain these pathways. Interestingly, the relationship between 2-oxoarginine reduction in the plasma of the subjects after FMT, potentially due to engraftment of these species, indicates a connection. As 2-oxoarginine is an intermediate in the arginine dehydrogenase pathway, we also investigated 4-guanidinobutanoate as this is present in the metabolon panel and also an intermediate in the arginine dehydrogenase pathway, which is implicated in L-arginine metabolism. A similar pattern is observed for 4-guanidinobutanoate. It has been shown previously that supplementation of L-arginine leads to a reduction in blood pressure,^43^ potentially via the production of NO, a potent vasodilatator.^44^ Hereby, there are several mechanisms in which a reduction in 2-oxoarginine may lead to a decreased blood pressure. First, as L-arginine serves as a substrate to produce 2-oxoarginine. Thus, a reduction in such metabolites after FMT could indicate a switch in metabolic pathways. Consequently, a preference for the conversion of L-arginine to L-citrulline and NO via nitric oxide synthase (NOS) may be favored over the production of 2-oxoarginine. Second, both 2-oxoarginin and 4-guanidinobutanoate possess a guanidine side chain. Generally, guanidine compounds have demonstrated potential as inhibitors of NOS,^45^ impeding the conversion from arginine to NO. However, these specific guanidine compounds have not been specifically tested and require further investigation. Lastly, these metabolites are recognized as uremic toxins,^46^ capable of inducing the generation of reactive oxygen species,^47^ which, in turn, is associated with hypertension.^48^ Given that the association is purely statistical, it is possible that the direction of the association may be different. For example, *C. aerofaciens* engraftment may lead to a reduction in blood pressure, which in turn could affect 2-oxoarginine levels.

From an alternative perspective, NOS is modulated by bilirubin. Initially, heme is transformed into biliverdin by heme oxygenase, followed by the conversion into bilirubin in the presence of biliverdin reductase.^35,36^ Bilirubin is an antioxidant, in which it can reduce reactive oxygen species, and thereby, reoxidize bilirubin into biliverdin.^49^ Intriguingly, while we discern a mechanistically plausible negative correlation between biliverdin and diastolic blood pressure, no significant correlations are observed between heme or bilirubin and diastolic blood pressure. This is to be expected given that bilirubin is a potentially toxic substance and the body has multiple mechanisms for its safe detoxification and disposition, including conjugation in the hepatocyte and thereby excretion into bile and urine.^50^

The results within this study should be taken in light of several limitations. As this is a combined cohort, including various FMT studies, the research question within each FMT study is slightly different. Thereby notable variations in donor subject choice and intervention type per sub-study were made, adding more complexity to the already heterogenous intervention. The potential confounding batch effects resulting from the aggregation of multiple sub-studies are likely overshadowed by other variables that influence engraftment and response, which in turn complicates the design of a randomized controlled trial due to various practical constraints. Additionally, the limited sample size further challenged the investigation. Lastly, we made the assumption that strains introduced resulted in the beneficial effect within the subjects. One could invert this assumption and suggest that disposition of native strains due to the FMT lead to restoration of homeostasis. This avenue was, however, not investigated. Despite the challenges, by applying this framework, we managed to generate plausible hypothesis and aid in further disentanglement of an FMT intervention.

## Conclusions

By utilizing a novel framework, we elucidated the complex and heterogenous FMT intervention, thereby establishing a connection between engrafted microbiota and clinical outcome parameters. A decrease in diastolic blood pressure observed in subjects receiving allogenic FMT is related to the engraftment of several strains such as the *C. aerofaciens*, *F. saccharivorans*, and *L. celerecrescens*. Engraftment of these strains concurrently seems to be linked to a decrease in intermediate metabolites from the arginine dehydrogenase pathway, including 2-oxoarginine and 4-guanidinobutanoate. These metabolites are known to be NOS inhibitors. By reducing the amounts of these metabolites, more NO can be produced, leading to more vasodilatation and ultimately lowering of the diastolic blood pressure.

## List of abbreviations

**Table:** 

FMT	Fecal Microbiota Transplantation
Met Syn	Metabolic Syndrome
NCEP	National Cholesterol Education Program
NO	Nitric Oxide
NOS	Nitric Oxide Synthase
rCCA	regularized Canonical Correlation Analysis
rCDI	recurrent *Clostridiodes difficile* Infection
SGB	Species Genome Bin

## Supplementary Material

Supplemental Material

## Data Availability

Raw metagenomic sequencing data of the Fatmed study has been uploaded to the European Nucleotide archive under accession number EGAS50000000409. The bioinformatics pipeline to process the metagenomic sequencing data and subsequently acquiring the taxonomic profiles, pathway abundances, and strain sharing rates is available at GitHub (https://github.com/EvdVossen/Metagenomic_pipeline/). The R-scripts (including R-markdown) containing the formal analyses and visualizations are available at (https://github.com/EvdVossen/MPS).
