# Supplementary figures and images for: Disentangle beneficial effects of strain engraftment after fecal microbiota transplantation in subjects with MetSyn
Source: Gut Microbes. 2024 Aug 20;16(1):2388295. doi: 10.1080/19490976.2024.2388295 (PMC11340759; doi:10.1080/19490976.2024.2388295)

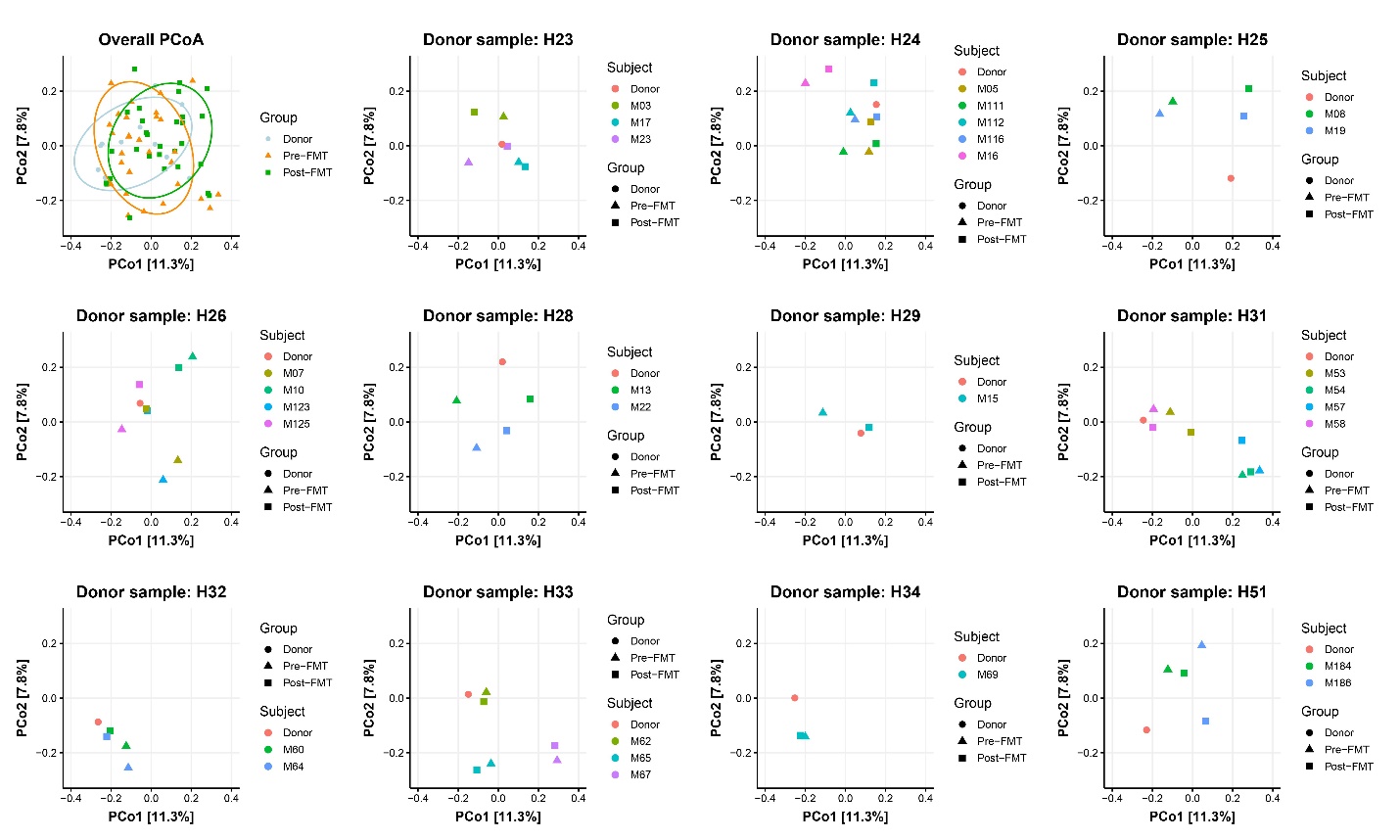
Fig. S1

Fig. S2


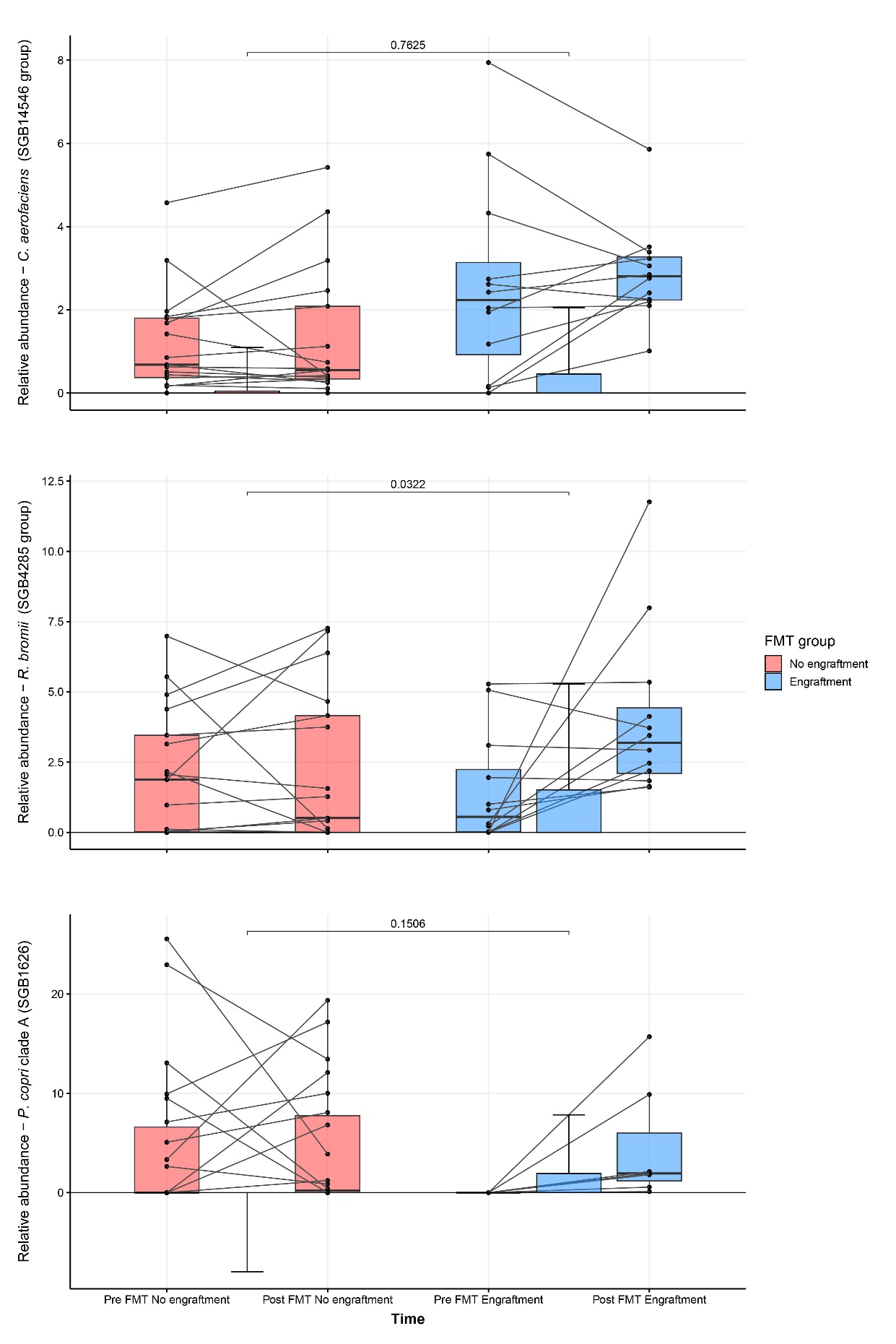


Fig. S3


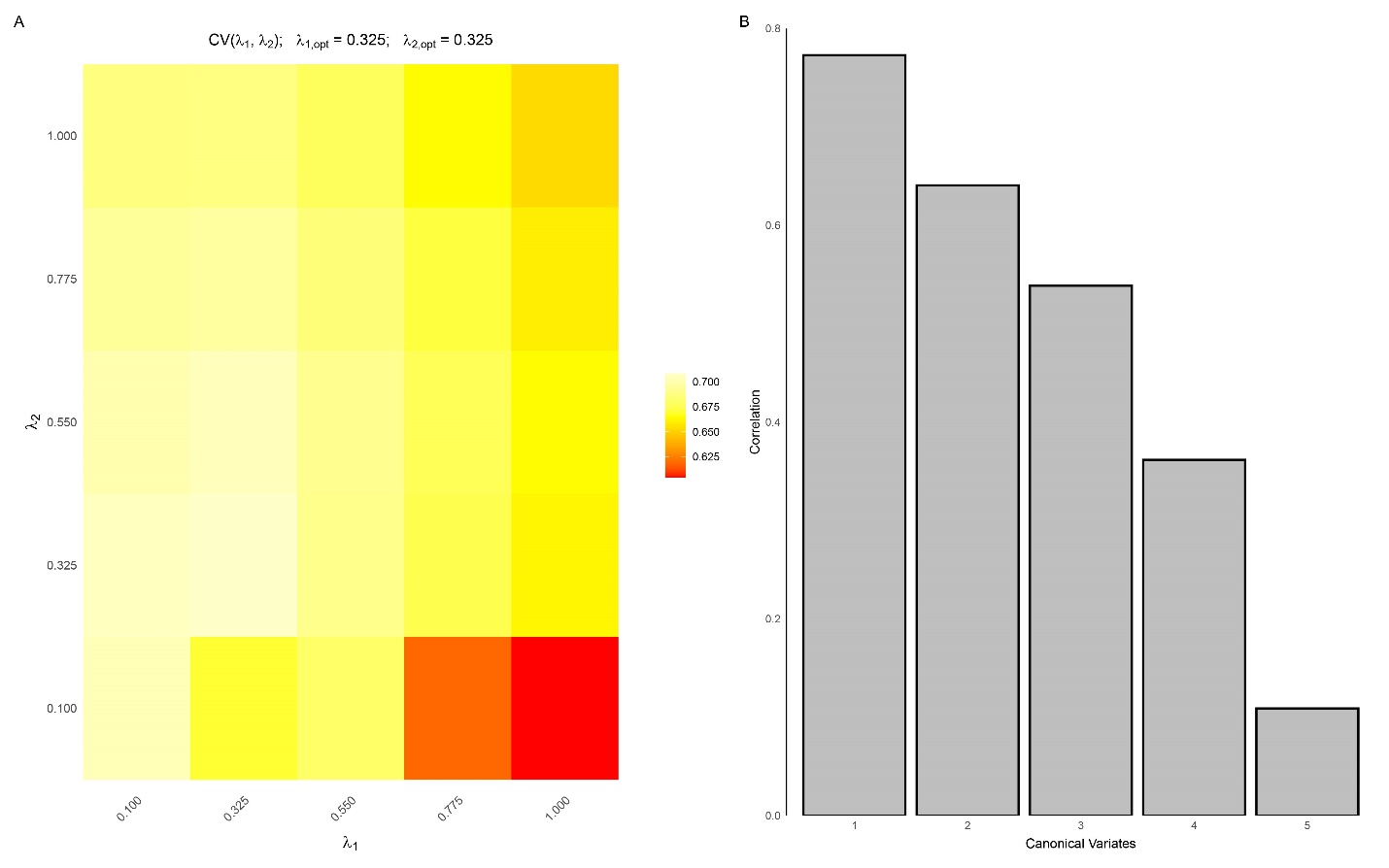


Fig. S4


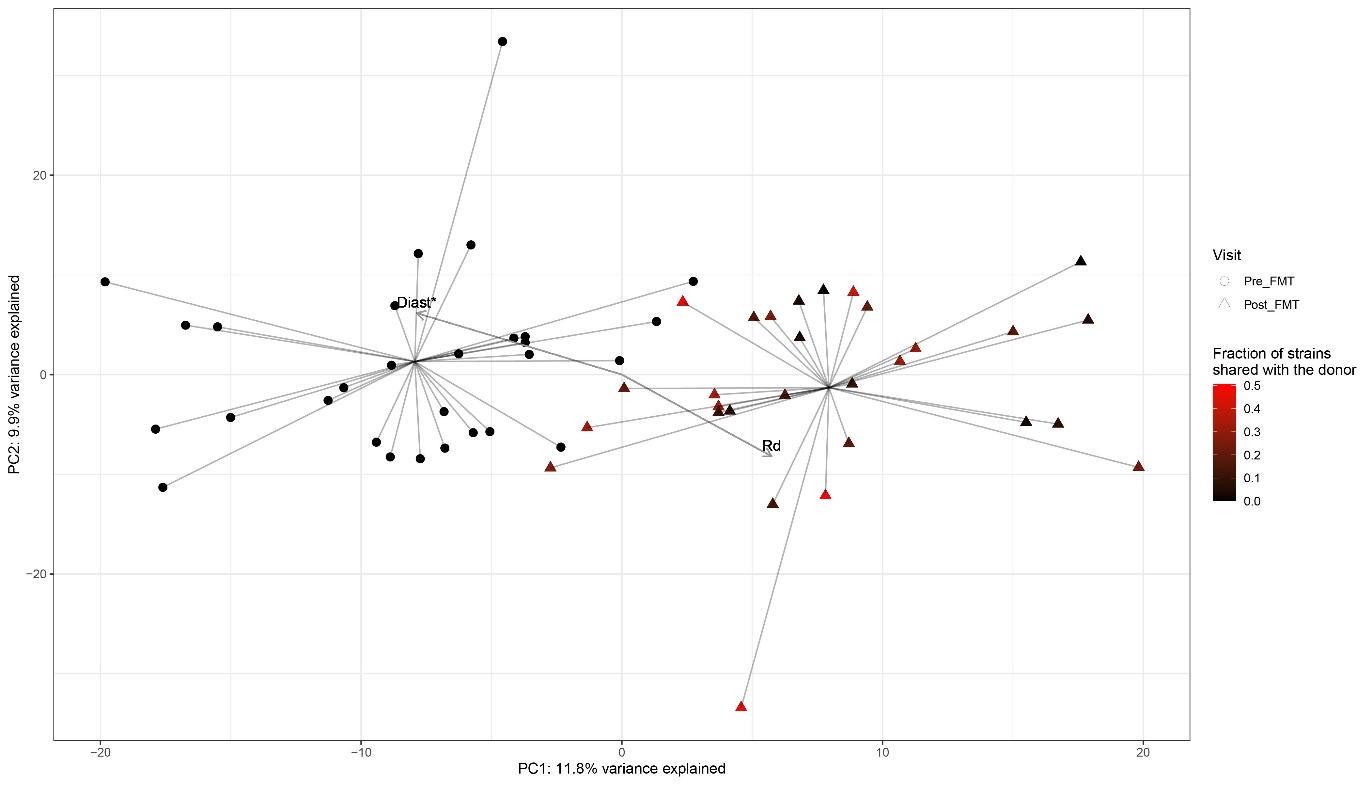


Fig. S5


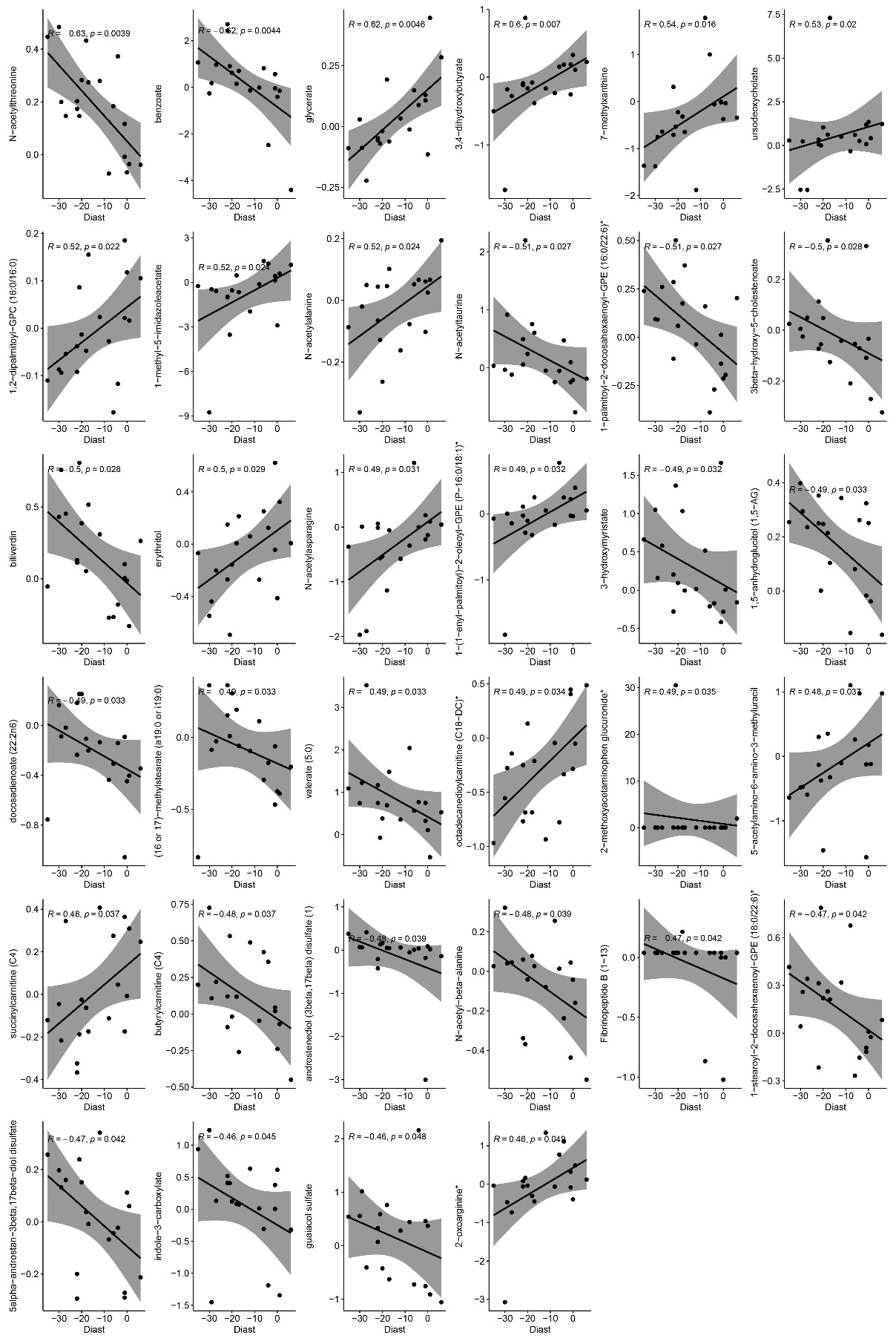


Fig. S6


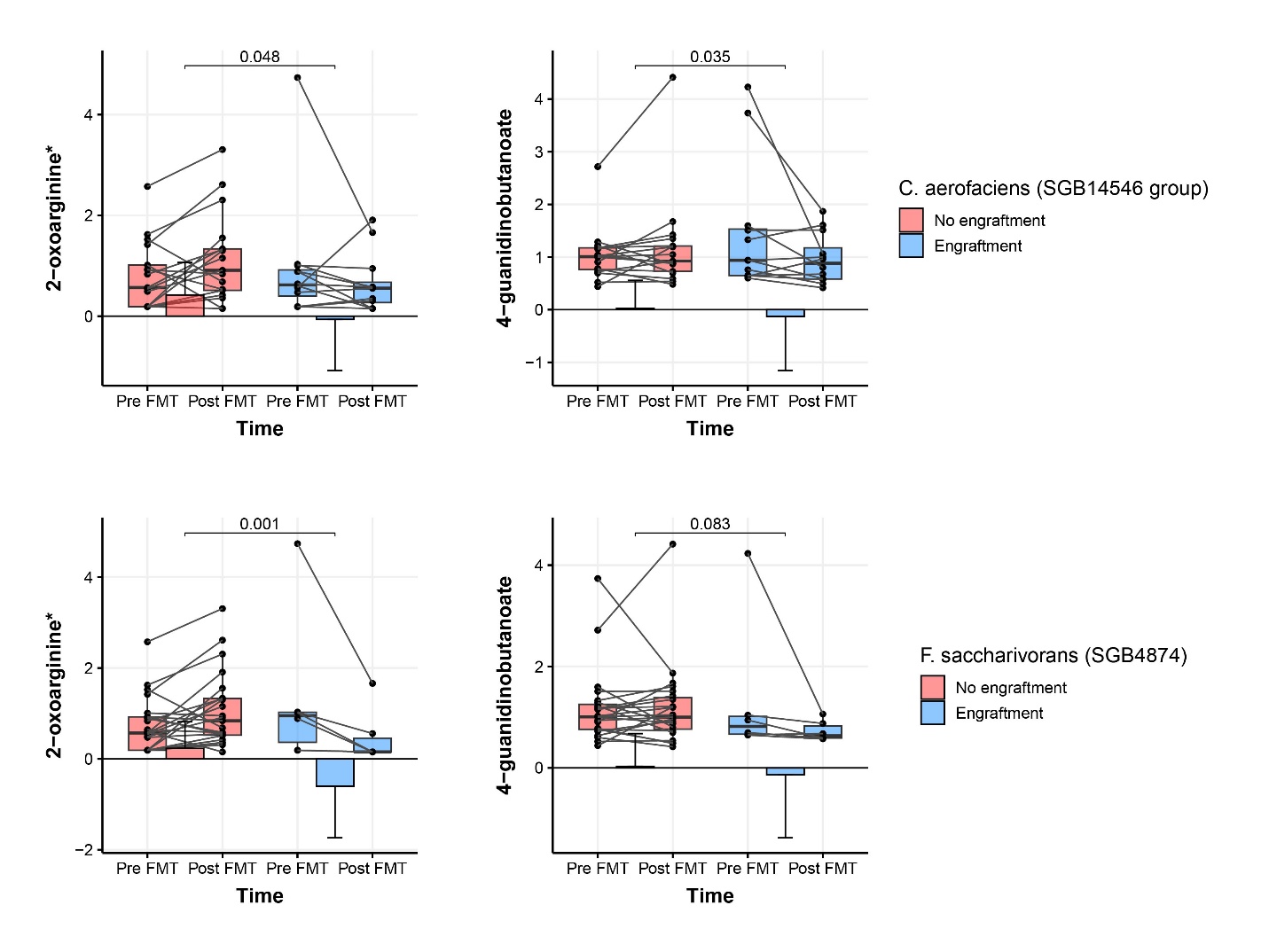

Supplement: Supplemental Material [file KGMI_A_2388295_SM8042.zip › Supp figures.docx]
